# Nutrient-Mediated Perception and Signalling in Human Metabolism: A Perspective of Nutrigenomics

**DOI:** 10.3390/ijms231911305

**Published:** 2022-09-25

**Authors:** Milan Kumar Lal, Eshita Sharma, Rahul Kumar Tiwari, Rajni Devi, Udit Nandan Mishra, Richa Thakur, Rucku Gupta, Abhijit Dey, Priyanka Lal, Awadhesh Kumar, Muhammad Ahsan Altaf, Durgesh Nandini Sahu, Ravinder Kumar, Brajesh Singh, Sunil Kumar Sahu

**Affiliations:** 1 Division of Crop Physiology, Biochemistry and Post-Harvest Technology, ICAR-Central Potato Research Institute, Shimla 171001, India; 2 Department of Molecular Biology and Biochemistry, Guru Nanak Dev University, Amritsar 143005, India; 3 Division of Plant Protection, ICAR-Central Potato Research Institute, Shimla 171001, India; 4 Department of Microbiology, Punjab Agricultural University, Ludhiana 141004, India; 5 Faculty of Agriculture, Sri Sri University, Cuttack 754006, India; 6 Division of Silviculture and Forest Management, Himalayan Forest Research Institute, Conifer Campus, Shimla 171001, India; 7 Department of horticulture, Sher-e-Kashmir University of Agricultural Science and Technology of Jammu, Jammu 181101, India; 8 Department of Life Sciences, Presidency University, 86/1 College Street, Kolkata 700073, India; 9 Department of Agricultural Economics and Extension, School of Agriculture, Lovely Professional University, Jalandhar GT Road (NH1), Phagwara 144402, India; 10 Division of Crop Physiology and Biochemistry, ICAR-National Rice Research Institute, Cuttack 754006, India; 11 College of Horticulture, Hainan University, Haikou 570228, China; 12 State Key Laboratory of Agricultural Genomics, BGI-Shenzhen, Shenzhen 518083, China

**Keywords:** nutrigenomics, human diet, hormones, mTOR, nutrient sensing pathways, epigenetics

## Abstract

The interaction between selective nutrients and linked genes involving a specific organ reveals the genetic make-up of an individual in response to a particular nutrient. The interaction of genes with food opens opportunities for the addition of bioactive compounds for specific populations comprising identical genotypes. The slight difference in the genetic blueprints of humans is advantageous in determining the effect of nutrients and their metabolism in the body. The basic knowledge of emerging nutrigenomics and nutrigenetics can be applied to optimize health, prevention, and treatment of diseases. In addition, nutrient-mediated pathways detecting the cellular concentration of nutrients such as sugars, amino acids, lipids, and metabolites are integrated and coordinated at the organismal level via hormone signals. This review deals with the interaction of nutrients with various aspects of nutrigenetics and nutrigenomics along with pathways involved in nutrient sensing and regulation, which can provide a detailed understanding of this new leading edge in nutrition research and its potential application to dietetic practice.

## 1. Introduction

Nutrients are essential organic substances, and how they are distinguished in the stomach is pivotal for stabilizing the body’s nutritional and energy requirements. Diverse gene expressions are elicited after food perception by their respective receptors [1]. Medical experts have recently become inquisitive about nutrition perception as it may open a new way to overcome many chronic diseases. In both rats and human subjects, recent developments in functional magnetic resonance imaging (fMRI) methods have granted non-invasive scanning of entire brain activity during the processing of information about ingested nutrients from the stomach depending on food consumption [2]. Several studies have suggested that the way food is perceived has stronger repercussions on metabolism and development in various species. Humans, for example, respond to the sight and scent of food by secreting chemical messengers (hormones) [3]. The diversity of sensory receptors plays a role in detecting taste, smell, pain, heat, and tactile mechano-sensory inputs. These sensory receptors are accommodated in specialized ciliated neurons, which perceive variation in ambient circumstances and assist in behavioural decisions ranging from food choices to avoiding dangerous circumstances [4]. Recent genetic research, on the other hand, recommends that sensory perception has other physiological functions, such as affecting energy homeostasis and lifespan by neural networks originating in sensory tissues. Mammalian cells possess diverse ways of sensing essential cellular nutrients such as glucose, amino acids, and lipids. Insufficiency in cellular nutrients in all organisms has significantly driven the need to have a detailed understanding of efficient nutrition signalling pathways.

**Figure 1:**
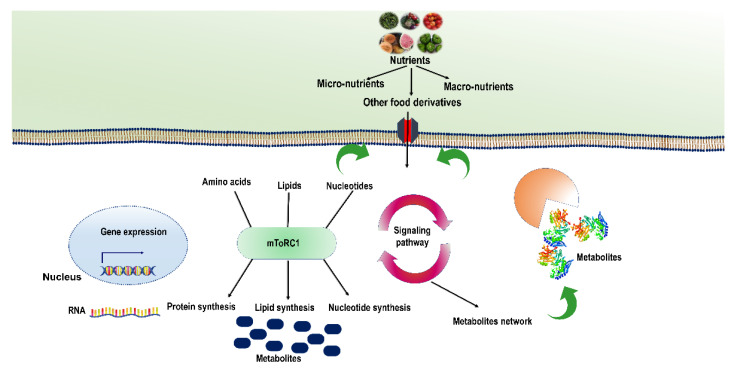
The main signalling pathways that regulate mTOR activity on the binding of micro- and macronutrients on their respective receptors on the cell membrane. Active mTOR plays the most vital role in cell growth and proliferation by stimulating diverse anabolic processes such as lipid synthesis, protein synthesis, and nucleotide synthesis.

Nutritional signalling is a broad term that refers to various cell signalling pathways influenced by nutrient availability. Signalling cascades are activated when nutrient levels change, modulating essential cellular activities including metabolism, proliferation, secretion, and autophagy. A cascade of signalling molecules that is highly conserved senses a particular nutrient and adjusts cellular physiology according to the availability of nutrients. This cascade includes TOR (target of rapamycin) kinase, PKA (protein kinase A), and AMPK (AMP-activated kinase) [5]. To synchronize cellular metabolism and biological operations, a multi-layered system evolved throughout time to detect variations in a large number of available nutrients and other metabolites. The most well-studied nutrient sensing and signalling networks to this date include AMPK and mTOR [6]. Nature has bestowed both unicellular and multicellular organisms with a complex and intricate toolkit in their cells through which they can easily detect and respond to a wide range of nutrients [7] (Figure 1). The lac operon efficiently gives sugar (glucose and lactose)-sensing techniques to bacterium cells. The lac operon cleverly uses sugar sensors and transcription factors to control the expression of genes encoding various metabolic enzymes [8]. In humans, the metabolic network is linked with diverse metabolites and advanced nutrient sensing systems for synchronizing various biological activities.

Metabolite perception and sensing machinery proved to be very significant for cells; through these mechanisms, cells can coordinate gene expression and signalling with cellular metabolism [9]. When the metabolite sensors present in the cells come in contact with a metabolite, with the assistance of a transducer (in most cases, acting as a signalling route) and the sensor protein, they transform the chemical messenger into cell signalling events. On the other hand, RNA and DNA are macromolecules that regulate signalling, cellular metabolism, and modification in gene expressions (epigenetics) [10]. Among other metabolites, amino acids, metal ions, purines, nucleotide derivatives, and SAM/ SAH bind to functional regions of RNA [11]. The metabolic behaviour varies across different cell types. As a result, different organelles use various metabolite sensing systems and facilitate inter-organelle metabolite signalling events. The biological importance of metabolite sensing may be better understood using a functional metabolomics approach [12]. In this review, we discuss detailed nutrient sensing mechanisms and signalling events by various metabolites (glucose, amino acids, and lipids), as well as the impact of nutrients on human health from a nutrigenomics perspective.

## 2. Nutrigenomics and Public Health

Genomics is based on information acquired from the Human Genome Project and other genomes, which resulted in a wealth of information on genes and their role in determining our health. The knowledge gained from these genomes has considerably improved our understanding of nutrition, diversity, and evolution of organisms [13,14,15,16,17,18]. The nutrition sciences are developing a relationship with the “omics,” disciplines bolstered by the Genome Project’s recent revelations, and new technical developments are coming at a breakneck pace. This research has spawned a new field of study known as nutrigenomics and notions such as “customized nutrition/diet.” Nutrigenomics is a branch of research that combines molecular biology, genetics, and nutrition. It is a relatively new and quickly expanding field of study. It focuses on the function of nutritional status or particular nutrients in the control of gene expression [19]. The growth of this subject and its application in improving food features and nutritional indicators will have far-reaching repercussions for the public health and nutrition industries. One of the outcomes may be the creation of tailored meals based on a person’s genetic code and focused on enhancing the expression of certain genes within it [20]. Traditionally, nutrition science has made the most practical contribution to public health by providing the best dietary guidelines for avoiding illness and supporting optimal health. Many public health hazards, such as cancer, diabetes, obesity, and other chronic illnesses (such as cardiovascular disease and hypertension), are impacted to some extent by hereditary factors. As a result, many dietary guidelines have been devised with the goal of improving the overall health of the community, as well as the health of persons who are at a high risk of developing these illnesses.

### Gene–Nutrient Interactions and Their Consequences on Health

The idea that what one eats affects one’s health is not new. Furthermore, it is generally recognized that persons have different nutritional requirements. Many studies have found that the environment and diet (both quality and quantity) are the essential factors determining one’s health and sickness. Many dietary components are bioactive, altering the genome, transcriptome, and proteome expression directly or indirectly, thereby regulating biological processes and supplying energy-generating substrates [21]. The human diet is considered to include over 20,000 compounds, with roughly 50 of them being essential for existence. Systematic investigations of macro- and micronutrient balance and turnover helped to approximate their daily requirements, which were used to determine recommended dietary allowances [22]. High-throughput genomics studies, such as single-nucleotide polymorphisms (SNPs), quantitative transcriptomics, proteomics, and metabolite changes, should be used in conjunction with bioinformatics to define nutrition [23]. Nutrigenomics refers to the use of technologies such as nutrigenetics (the study of DNA, including SNPs), transcriptomics (mRNA), proteomics (protein complement), and metabolomics (metabolite complement) in the context of nutrition [24]. A nutrigenomics-based personalized diet may improve public health by incentivizing people to modify their eating habits. The authors of [25] studied the impact of genetic feedback on obesity risk in the behaviour of people of normal weight (*n* = 249). People who were warned about their increased risk of obesity were more motivated to consume a healthy diet. Nutrients and meals normally interact with genes in a positive way; however, this interaction may occasionally turn deadly, which needs to be understood in detail. Both environmental and genetic variables impact humans, and both must be taken into account to maintain normal health status. Nutrition science looks at how nutrients help to keep the body’s homeostasis in check at the cellular, tissue, and organ levels. This research must understand the mechanism of nutrient-dependent interactions at the genetic, molecular, protein synthesis, and metabolic profile levels. As a result, nutrition research has progressed beyond epidemiological and physiological elements to molecular biology, genetics, and nutritional genomics. Table 1 depicts several examples of gene–nutrient interactions and their health implications.

## 3. Dietary Supplements and Fortified Food

The best foods provide appropriate health support. Certain foods, such as breakfast cereal, already have vitamin and mineral supplements, and there is a rising range of superfoods purported to have health-promoting properties. Nutraceuticals are natural products that are beneficial for health or for preventing diseases. One study demonstrated the ability of dietary peptides to inhibit angiotensin-converting enzymatic activity [43]. Despite promising preclinical data, human clinical trials are yet to be conducted concerning the effects that milk peptides have on human blood pressure [43]. An integration of putative human trials with nutrigenomic technologies can decipher how these nutritionally produced peptides differ from synthetic angiotensin-converting enzyme inhibitors [44]. Patients suffering from osteoarthritis could perhaps benefit from supplements such as chondroitin and glucosamine. Researchers have demonstrated the disease-modulating effects of glucosamine sulphate and symptomatic improvements [45,46]. However, concerns about bias, usefulness, and toxic effects remain problematic [47,48]. Additional evidence is necessary to outline the benefits of this programme. Nutraceuticals have shown promise in recent clinical trials, but quality control and scientific research must be performed to fully assess the effectiveness of these drugs. Owing to the wide range of alleged nutritional substances present in products in the food industry, dietary supplements, and herbal industries, products containing these compounds are called “nutraceuticals.” Although certain food substances could perhaps meet the criteria for health claims based on their functionality within human bodies, they are not quite as strictly controlled as drugs, which inevitably leads to considerations regarding routine, long-term use. The “European Commission” issued a proposal addressing food supplements in the EU White Paper of 14 January 2000. This will synchronize the regulations for vitamin and mineral supplements, marking them as functional foods. These initiatives could signal the first leap toward more complete and accurate toughening of legislative changes, as it is recommended that future proposed changes could be designed to cover products that contain other nutrients or additives. Functional genomics tools will allow researchers to precisely analyse the impact of dietary supplements, particularly nutraceuticals, on gene expression and cell function worldwide. The same principles employed in assessing chemicals can also be used to analyse genetically modified foods. To be successful, innovative food items with health benefits must be perceived as a positive choice. Products with the highest probability of success are new foods with appealing aesthetics and health benefits that customers are eager to understand. Just one strategy to guarantee product design reviews involves consumers. New food product marketing that does not have a straightforward benefit to consumers is detrimental to nutrition research and the food processing industry.

## 4. Nutrients as a Signal in Human Metabolism

The sensing of nutrient signals via cellular receptors and molecules is the key factor for healthy human metabolism. Any perturbance in the sensing and signalling of nutrients can cause metabolic diseases. Most cellular processes have evolved because of the scarcity of nutrients. Hormone signalling plays a crucial role in the detection of extracellular and intracellular metabolites such as lipids, sugars, amino acids, etc. Hormones are the long-range signals facilitating response coordination at the organismal level. Many different mechanisms are involved in sensing different nutrients in mammalian cells.

### 4.1. Amino Acid Signalling

Amino acids are proteins’ major constituents and are regarded to be the building blocks of proteins. Amino acid signalling plays a crucial role in protein biosynthesis, which is quite expensive in energy utilization. One of the interesting conditions is during starvation or nutrient limitation: proteins themselves act as reservoirs for amino acids, which are degraded via the proteosome-mediated degradation pathway and autophagy for re-utilisation of the amino acids. These amino acids act as a source for the generation of glucose via gluconeogenesis or ketone bodies. The availability of amino acids is strongly regulated by serine/threonine kinase and TOR (a cell growth modulator), which serve as molecules and receptors for amino acid signalling. Lysosomes are the key players in amino acid signalling tom TORC1. GCN2 is another group of kinases known to be involved in sensing amino acids and leads to the expression of stress-responsive genes under nutrient-limiting conditions [49].

### 4.2. Glucose Sensing and Signalling

Glucose is the key requirement for every organism, which is physiologically maintained in a narrow range via various means. There is a tight regulation in glucose intake, storage, and utilisation mechanisms at different levels. AMPK is known to be involved in modulating glucose and glycogen metabolism through different mechanisms, such as the upregulation of glucose uptake by increasing the expression of glucose transporters [50], the promotion of glycolysis during anaerobic conditions, the inhibition of gluconeogenesis, and the promotion of mitochondrial biosynthesis. During glucose starvation, AMPK induces autophagy, a process by which cells cope with stress conditions [51]. Along with AMPK, mTORC1 plays an essential role in intracellular energy control via different pathways and mechanisms. GCK or glucokinase is another important kinase that is active only in glucose abundance and is involved in glucose homeostasis. GCK is the most abundant hexokinase in the liver, in which glucose levels are maintained in scarcity via gluconeogenesis and in abundance through glycogen synthesis.

### 4.3. Lipids Signalling

Lipids are a class of nutrients characterized by hydrophobic carbon chains that act as energy sources and play a fundamental role in membrane biosynthesis and integrity. Lipids play a role in signal transduction. Phosphoinositides (PPIns) are lipid signalling molecules that act as master regulators of cellular signalling and, therefore, are essential for cell growth, death, and metabolism. PPIns can regulate integral membrane proteins. The synthesis and recruitment of these PPIns are achieved in a regulatory manner by two master regulators named phosphoinositide kinases and phosphatases. The downstream regulation of these enzymes leads to the production of phosphatidylinositol 3,4,5-trisphosphate (PIP_3_), mainly associated with numerous human diseases, development disorders, immune disorders, unbalanced enzyme activities, etc. [52]. Lipid metabolism is regulated by AMPK, which decreases fatty acid synthesis and thus inhibits lipogenesis and increases fatty acid oxidation.

### 4.4. Fatty Acid Signalling

Fatty acids are a source of energy and are synthesized from triglycerides; they function as signalling molecules regulating various cellular processes and functions according to chain length. Free fatty acids from dietary fibres contribute to key physiological processes regulated through receptor-mediated signalling, which depends upon carbon chain length. G-coupled protein receptors (GPRs) are involved in FFA signalling. The short-chain fatty acids (SCFAs) have six carbons, including acetate, butyrate, and propionate, produced by the fermentation of fibres by gut microbiota [53]. SCFAs can activate GPR41 and GPR43, whereas long-chain fatty acids (LCFAs) can activate GPR40 and GPR120. SCFAs are histone deacetylase inhibitors (HDACs), which play a role in chromosome structure modification and gene expression regulation. Therefore, SCFAs also participate in signalling via HDAC-mediated pathways [54]. FFARs (Free fatty acid receptors) signalling in diverse metabolic processes can provide insights for nutritional science and drug discovery [55].

### 4.5. Bile Acids and Cholesterol as Signalling Molecules

Bile acids are also known to be signalling molecules and act as metabolic regulators that lead to the activation of nuclear and G-protein coupled receptors (GPCRs). GPCRs are known to be involved in energy homeostasis, glucose, and lipid homeostasis. The conversion of cholesterol strongly maintains cholesterol homeostasis in bile acids, which keeps the liver healthy. Perturbance in bile acid metabolism leads to several disorders, such as cardiovascular diseases, chronic liver infections, diabetes, etc. [56]. Cholesterol biosynthesis regulation occurs in close proximity to cholesterol sensing. In the presence and absence of cholesterol, the SCAP/SREBP (SREBP cleavage activating protein/Sterol regulatory element-binding protein) complex plays a role in the endoplasmic reticulum and Golgi apparatus [57].

## 5. Receptor-Mediated Nutrient Sensing in Metabolism

### 5.1. Nutrient-Sensing Mechanisms

The ingestion of certain nutrients may stimulate the secretion of hormones or other signalling molecules in the bloodstream. It is necessary to measure cholesterol levels to avoid activating the energetically challenging cholesterol biosynthesis. In the event of an amino acid deficit, cellular proteins are used to retain essential components. The pathways for the two proteins function together to keep blood cholesterol in check [58]. The sensing process can be either directly binding with the macro- or micronutrient towards its receptor or indirect mechanisms resulting from the identification of a metabolite that indicates the abundance of the nutrient [30]. In the basic catabolic pathway, cellular components are broken down into useable substances. The lysosome is a tiny space where nutrients are harvested. This process allows survival by preserving cellular energy and vital functions. Plasma membrane protein GLUT2 (encoded by the gene SLC2A2) is a transporter with a rather low affinity for glucose. GLUT2 acts as a true sensor for glucose since it is only active at high but not at low glucose concentrations [59]. GCK (glucokinase) is the enzyme that catalyses the first step in glycogen synthesis and glycolysis. When blood glucose levels are very low, GCK expels non-phosphorylated glucose from the liver and muscles and transfers it to the brain [60].

### 5.2. Dietary Signals and Sensors

Gene expressions are synchronized with the type of nutrients one takes by virtue of its binding to transcriptional regulators. Most nutrient regulators seem to be in the transcription factor (TF) superfamily (nuclear receptors). Nutrients, including their derivatives, are associated with such receptor superfamilies [61,62]. During ligand binding, nuclear receptors undergo a conformational change to allow co-activator recruitment to activate transcription. A nutrient sensor alters the level of DNA transcription in metabolically active tissues. Nuclear receptors control many important processes, particularly nutrient metabolism, cell proliferation, as well as cell division. Activating the hormone receptors also affects a wide variety of cell activities. The peroxisome proliferator activator receptor-α (PPAR) group of nuclear receptors serves as nutrient sensors and regulates the expression of particular genes [63]. These target genes are involved in a multitude of metabolic processes, such as oxidation (fatty acids), ketone body formation, glucose synthesis (from non-carbohydrate precursors), etc. [63]. PPAR is essential during fasting when adipose tissue releases free fatty acids (FFAs). FFAs are partially and/or fully oxidized in the liver. These fatty acids bind PPAR, modulating the genetic expression by binding to their promoters [63,64,65]. As PPAR agonists, elevated fatty acids function through an enhanced PPAR signalling, which has a gluconeogenic and glucose-stimulating influence. Research linking obesity and type 2 diabetes may benefit from a better understanding of the PPAR function in type 2 diabetes [65]. Fat deposition around the viscera may be linked to FFA levels. This class of molecules can act as hunger or glucose signals requiring attention. Increased gluconeogenesis is likely in this case. A substantial portion of gene regulation involves co-repressors and co-activators. TFs congregate around clusters of co-activators, allowing them to increase DNA transcription. These studies demonstrated the possibilities in nutrigenomics and genomics research and how they can be applied to research the ways in which different people express different gene patterns based on their diet type [66].

### 5.3. Nuclear Receptors as Nutrient Sensors

Although some molecules are assumed to stay outside of cells, their ability to work once outside of the cell is thanks to this fact. The role of nuclear receptors is to transduce external stimuli and then regulate gene expression. This nuclear receptor family includes 48 members, each of which fulfils roles within the cell and the organism [67]. Both macronutrients and metabolites are nucleolar ligands. Nucleic acids can be regulatory molecules for nuclear receptors or DNA binding domains. The number of genes that can be activated by a nuclear receptor, ranging from about 100 to 1000, is increased when a co-activator protein appears. Nuclear receptor superfamily members regulate many physiological functions. Low-molecular-weight lipophilic compounds control the expression of these transcription factors. Nuclear receptors and ligands are critical in treating illnesses because they play a significant role in homeostasis maintenance [68]. More natural nuclear receptor ligands in the human diet need to be explored; as a result, opioid pain medication can no longer be used as a treatment choice.

### 5.4. Metabolism and Nutritional Evolution

Life must abide by thermodynamics laws to survive, meaning that perhaps a constant supply of energy is necessary for the survival of an organized system. Our nutrition provides us with adequate nutrients essential to sustain our body’s energy, reproduction, and development [69]. Food intake is regulated by hedonic sensation and homeostatic function. The body uses macronutrients for energy as they are metabolized. The abundant energy-rich nutrients are retained in the liver and muscles. Our bodies use stored fat to survive seasonal famine. Fats help to improve running stamina, which is the evolutionary foundation for predatory behaviour. However, due to modern foodstuffs and the sedentary lifestyle, humans no longer need to find sources of food, such as finding animals and searching for them [70]. There are numerous ways to obtain healthy nutrition, but the results of the food we consume are often complex. A balanced diet, such as that of the Mediterranean or Nordic, must be remembered. Our diet continually interacts with the genomes that control our metabolic organs. We can protect ourselves from illness by eating well and staying busy. The industrial revolution has led to a lack of activity (exercise) needed for employment and transportation. This has also affected the amount of sugar, fibre, and fat content in diets, giving a higher energy density and glycaemic load. Diets are high in sugar and fat, which are good for energy, but low in fibre. The “energy flipping point” has already happened in high-income countries and is now hitting every community worldwide [71].

### 5.5. Epigenetic Signalling and Intermediary Metabolism

Numerous genes influenced by food components and energy status are all implicated in health and disease [72,73,74,75]. For multiple-gene disorders, such as some chronic illnesses, diseases such as diabetes and high blood pressure are polygenic, which means that they are the product of several genes and gene variants. Two models have been used to study nutrition/genetics interactions. First, a food additive is studied in detail, utilizing existing molecular and cell biology techniques [76]. Second, high-throughput technologies have allowed investigators to examine numerous genes simultaneously [77,78]. This work is also advancing nutrigenomics and genetics. At first, the spotlight of genome-wide analysis was short DNA sequences of mRNA and protein-coding regions (mRNAs). Progress in sequencing has enabled the evaluation of the entire genome, thus providing a better picture of the non-protein-coding RNA that regulates gene expression [79]. Recent advances in molecular sequencing technology have led to a revolution in understanding developmental and disease regulation [80,81]. As opposed to the methodologies used for the Human Genome Project, advanced sequencers are 50,000-fold faster and have substantially cut the price of DNA sequencing by a factor of even more than about 50,000. New technologies are facilitating significant improvements in research focused on nutrition, the relationship between nutrition and health, and the effect of epigenetics on health and disease [79].

### 5.6. Circadian Control of Metabolic Processes

Human circadian patterns include a diurnal sleeping pattern and a circadian eating pattern. Molecular clocks in the cell nucleus control circadian rhythms, which are implemented by a network of transcription factors that are all expressed in the cell nucleus [82]. It is important for metabolic pathways to have an accurate and synchronized circadian clock. The negative effects of inadequate sleep, such as late-night feeding, night-time lighting, time-zone shifting, and spatial disorganization, are evident in many people. This hampers the metabolic process [83], and researchers have found that up to 15% of all genes or transcripts are expressed in a tissue or organ according to a circadian rhythm [84]. The circadian clock can also be regulated by how much energy the cell has taken in as well as how much energy the cell has released by cellular metabolism. The AMPK sensor combines a biochemical energy source (such as glycogen) and a food reservoir (such as protein) into an internal clock. This inhibition of the circadian clock is due to the interaction between circadian and metabolic signalling [85].

### 5.7. Modulating the Circadian Clock by Metabolic Systems

Proteins that control circadian rhythms are expressed in a wide variety of peripheral tissues, including the liver. The entrainment of the clock to food includes glucocorticoid signalling, temperature via HSF1, and ADP-ribosylation. In this fashion, the circadian clock synchronizes daily behavioural cycles of sleep–wake, fasting–eating, and exercise with the body’s anabolic and catabolic processes. The central and peripheral clocks are also synchronized through post-translational modifications of transcription factors (TFs) and histone proteins that control the expression of genes that stem directly from changes in the level of metabolites [86]. Therefore, mammals and other animals use mechanisms such as redox flux, NAD+ oscillation, ATP supply, and mitochondrial function to control the post-translational modifications. For example, the redox-based clocks reflect a rhythmic oscillation in the redox state of the family of peroxiredoxin antioxidant enzymes that produce reactive oxygen species (ROS). Further, NAD+ acts as an electron shuttle during catalysis and participates in other enzymatic functions as well [87].

## 6. Nutrient Sensing and Regulation

Nutrients are simple organic molecules exploited in biochemical processes that result in energy production, and they are important constituents of cellular biomass. Organic compounds such as glucose and other related sugars, amino acids, and insulin or growth molecules are prime cellular nutrients, and diverse mechanisms to sense their richness are functional in mammalian cells [7]. Considering the significance of nutrient homeostasis for a healthy body environment, it is surprising that limited data on direct nutrient sensing mechanisms are present in the public domain. The regulation between gene expression outputs and cellular metabolic state is very crucial for the cell. Cells, being a smart entity of the body, have evolved to create a refined sensor and signal transduction system to detect the availability of diverse nutrients and regulate the cellular processes to accomplish their bioenergetic demands. Some nutrients may bind directly to their respective receptors or others indirectly through the detection of secondary molecules that depict nutrient abundance [7]. Both single and multicellular organisms exhibit an essential property, i.e., sensing diverse nutrient fluctuation from the surrounding ecosystem and their regulation, accordingly. This property encourages cells to survive under nutrient scarcity and proliferate during their abundance.

**Figure 2:**
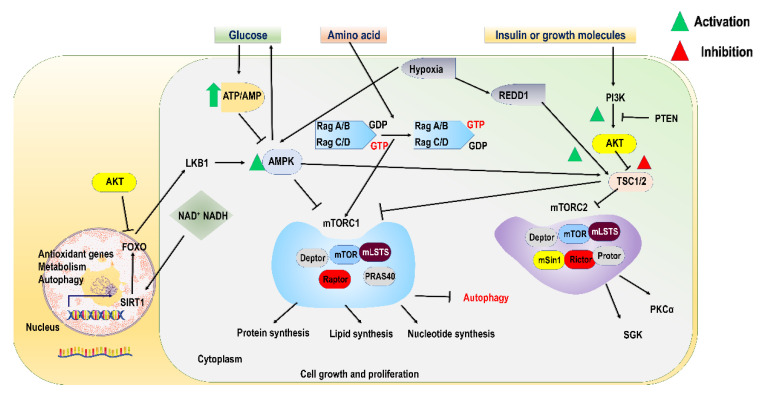
Nutrient signalling to mTOR: Cells possess a sensing system that senses nutrients and metabolites for proper communication and the regulation of cell signalling and metabolic status. mTOR exists in two distinct multiprotein complexes, termed as mTOR complex 1 and mTOR complex 2. mTORC1 is the main one, which plays an important role in sensing diverse nutrients and merging signals from metabolites and micro- and macronutrients to link anabolic processes with nutrient availability. It regulates the activity of various kinases through phosphorylation The molecular mechanism behind the effect of growth factors, glucose, amino acids, cellular energetic status, and hypoxic stress conditions on mTOR activation are fully explained. REDD1: regulated development and DNA damage responses 1; PI3K: phosphatidylinositol 3-kinase; TSC1/2: tuberous sclerosis complex ½; Deptor: DEP domain containing mTOR-interacting protein; mLST8: mammalian lethal with SEC13 protein 8; Raptor: regulatory associated protein of mTOR; Rictor: raptor independent companion of mTOR; PRAS40: Akt/PKB substrate 40 kDa.

At the cellular level, nutrients may act as a ligand for receptors of various transcription factors and can be utilized by distinct pathways, thereby changing the concentration of substrates or intermediates. Nutrients affect signalling pathways both positively and negatively. Cells withstand or balance nutrient variability by precisely synchronizing anabolic processes with catabolic ones [88]. The mTOR (mechanistic target of rapamycin) signalling pathway is in the middle of this synchronizing action. The mTOR pathway is activated in a nutrient-enriched environment and signals cell proliferation by activating anabolic processes that involve the synthesis of macromolecules (proteins, amino acids, fatty acids, and nucleotides) and by suppressing catabolic processes causing autophagy (Figure 2) [89]. Contrarily, nutrient inadequacy suppresses anabolic processes by inhibiting the mTOR pathway, whereas catabolism is stimulated to generate adequate energy, and nutrient molecules to regulate the fewest biological processes crucial for survival [90]. Therefore, dysregulation in the mTOR system coordinating such responses generally triggers metabolic disturbances and leads to various human diseases, including cancer and diabetes [91,92]. Therefore, a complete understanding of the mTOR pathway and its regulation by diverse nutrients at the molecular level is very crucial. In addition, it is also important to discover how the cell regulates its growth and survival under low/high-nutrient environment. Based on the current understanding, we have discussed the molecular mechanisms behind the regulation of mTOR system by diverse nutrient molecules (e.g., glucose and amino acids).

### 6.1. Role of mTOR Complex in Nutrient Sensing

mTOR kinase plays a crucial role in the cellular sensing of diverse nutrients by phosphorylating serine/threonine amino acids belonging to the PI3KK (phosphatidylinositide-3 kinase related kinases) superfamily and exists in two distinct multiprotein complexes, viz. mTORC1 (mTOR complex 1) and mTORC2 (mTOR complex 2). These complexes have overlapping yet different functions, regulations, and susceptivity to rapamycin [93]. mTOR kinase plays a vital role in the cellular sensing of diverse nutrients through the PI3K/AKT (phosphatidylinositol 3-kinase) pathway. The binding of insulin or growth molecules to cell receptors stimulates PI3K, which leads to the activation of AKT and mTORC1 through the suppression of tuberous sclerosis complex 1/2 (TSC1/2) (mTOR inhibitory proteins) [94]. Macromolecules such as glucose and amino acids have been identified as potent stimulators of the mTORC1 complex consisting of Deptor (DEP domain containing mTOR-interacting protein), mLST8 (mLST8 (mammalian lethal with SEC13 protein 8), Raptor, and PRAS40 (Akt/PKB substrate 40 kDa) [95] (Figure 2).

Raptor (regulatory associated protein of mTOR) protein is very significant for mTORC1′s functions because it employs substrates for phosphorylation [96]. Debtor and PRAS40 emerge to be both substrates and suppressors of mTORC1 and play no role [97,98]. The two most characterized mTORC1 substrates comprising ribosomal S6 kinase (S6K) and eukaryotic translation initiation factor 4E binding protein (4E-BP) carry most of the activities regulated by mTORC1 [99].

On the other hand, mTORC2 comprises mSin1, Protor, mLST8, Deptor, and Rictor (Raptor independent companion of mTOR). mTORC2 regulates the activity of various kinases, i.e., Akt, SGK, and PKCα, through phosphorylation of their hydrophobic segments [100] (Figure 2). Rictor plays a role similar to Raptor in recruiting substrates for phosphorylation in mTORC2, thus clearly describing the different choices of substrates by both mTOR complexes [101]. mSin1 and mLST8 proteins are critical for the coherence and catalytic activity of mTORC2 [102]. The function of Protor is currently unknown [103]. Unlike mTORC1, mTORC2 is unable to sense rapamycin, although in a few cells, the continuous exposure of rapamycin for a long time can suppress mTORC2 indirectly by inhibiting mTORC2 assembly [104].

### 6.2. Mechanisms Underlying Nutrient Signalling and Regulation

The mechanism of mTORC2 regulation is not clearly established; however, the available literature is understandable through some complex pictures of the mechanism for mTORC1 regulation. Diverse nutrients (amino acids, glucose, and growth factors) act as signalling molecules and play roles in the regulation of mTORC1.

#### 6.2.1. Role of Growth Factor as Signalling Agent for Regulation of mTORC1

Amid the various upstream signals, the regulation of mTORC1 by diverse growth factors has been fully studied (Figure 2). Growth factors act as signal agents and attach to appropriate receptors (tyrosine kinases), which stimulate the PI3K-AKT pathway [105]. This pathway involves phosphorylation and suppression of the TSC1/2-TBC1D7 ((Tre2-Bub2-Cdc16)1 domain family member 7) complex through phosphorylated AKT, which ultimately leads to the activation of mTORC1 [106]. This complex increases the inherent GTPase activity of Rheb by working as GAP (GTPase activating protein) for the Rheb [107]. The attachment of GTP makes Rheb active, hence making it crucial for mTORC1 activation. Therefore, growth factors act as potent signalling molecules by increasing the Rheb–GTP-to-Rheb–GDP ratio and suppressing the TSC2 GAP activity for mTORC1 activation (Figure 2). The suppression of in vitro GAP function of TSC2 by phosphorylation remains less understood due to the inadequacy of definite solid evidence. More apparently, the dissociation of the TSC1/2 complex takes place by phosphorylation of TSC2, which leads to its dislocation from the Rheb region, thereby triggering mTORC1 activity by increasing Rheb–GTP.

#### 6.2.2. Role of Glucose as Signalling Agent for Regulation of mTORC1

Most of the anabolic processes in the human system require ATP as a source of energy to carry out enzymatic reactions for the generation of biological macromolecules (lipids, nucleotides, and proteins) for cell growth and division. In actively dividing cells, the main energy source is glucose, catalysed through a series of enzymatic reactions during glycolysis [108]. There is a generation of two ATP molecules from the overall glycolysis cycle, whereas thirty-six molecules of ATP are produced per glucose molecule during aerobic respiration. Reduction in intracellular ATP levels causes a rapid reduction in glucose concentration, which leads to a subsequent increase in both AMP/ATP and ADP/ATP ratios. A serine/threonine kinase, i.e., AMPK (AMP-dependent kinase), senses an increase in both ratios, mainly the AMP/ATP ratio, and is considered an important sensor of cellular energy in all eukaryotes [109]. AMPK consists of α (catalytic) and βγ (regulatory) subunits and is activated upon the attachment of ADP or AMP to the γ subunit and by LKB1 (serine/threonine kinase) through phosphorylation at threonine 172 residue [110]. When quite low, the AMPK/LKB1 pathway senses the energetic status of cells and stimulates glucose uptake and leads to mTORC1 suppression. AMPK becomes activated under a nutrient-poor environment and passes stress signals through phosphorylation of TSC2 and Raptor genes to mTORC1. Inhibition of mTORC1 takes place via phosphorylation of TSC2 at serine residue by AMPK, stimulating its GAP activity, which leads to the suppression of Rheb [111]. Cellular energetic stress due to hypoxic conditions also brings about the deactivation of mTORC1 by involving the AMPK-TSC2 pathway. Under hypoxic conditions, the regulated development and DNA damage responses 1 (REDD1) gene’s expression is induced, which in turn deactivates mTORC1 by involving the TSC-dependent pathway [112] (Figure 2). mTORC1 can be deactivated without depending upon TSC by AMPK through direct phosphorylation of Raptor [113]. The studies depict that AMPK plays an important role as the main system for measuring inputs generated through energetic stress, which causes an inhibition of mTORC1 activation through both TSC-dependent and independent pathways. Higher levels of NAD^+^ and NADH create oxidative stress in the cell, which causes the activation of antioxidant genes by stimulating FOXO proteins expression, as shown in Figure 2. SIRT1 senses an increase in NAD^+^/NADH levels and plays a role in the activation of FOXO proteins [114].

#### 6.2.3. Role of Amino Acids as Signalling Agents for Regulation of mTORC1

Several studies have indicated that amino acids act as signalling agents for mTORC1 activity in uni- and multicellular organisms. Hall and colleagues’ work on budding yeasts proved amino acids’ role in mTORC1 activity [115]. Similar work on mammals has shown a connection between mTOR and amino acids, where rapid suppression of mTORC1 occurs because of amino acid depletion in CHO-IR cells [116]. Exploring the molecular mechanism behind the role of amino acids in the signalling to mTORC1 is an important area of research. Rag GTPases are considered important mediators in amino acid signalling to mTORC1. Studies have demonstrated the role of Rag GTPases as important mediators of amino acid signalling to mTORC1 through biochemical analysis [117]. Rag GTPases consist of four evolutionarily well-conserved Rag members (RagA, RagB, RagC, and RagD) that are present throughout all eukaryotes. Among the four Rag members, two (RagA and RagB) are interrelated with respect to their amino acid sequence and are the same as yeast Gtr1p, whereas the other two (RagC and RagD) are identical to yeast Gtr2p but are functionally less important. All four Rag members congregate in heterodimeric forms, resulting in four different dimer formations. The crystal structure of Gtrp1–Gtrp2 yeast was recently analysed, revealing that Rag protein includes two domains, i.e., C- and N-terminals. These Rag domains are functionally very crucial, as the C-terminal plays an important role in the formation of stable dimeric structure, whereas the N-terminal is pivotal for the activity of GTPase. Studies conducted on Rag mutants reported that Rag A/B (dominant negative mutant form), if not bound to GTP, can suppress mTORC1 activity even in the presence of the high number of amino acids. On the other side, the GTP-bound form of RagA/B (dominant active mutant) was enough to control mTORC1 activity even under complete depletion of amino acids. The expression of the Rag C/D mutant did not cause any significant effect on mTORC1 activity. Through these studies, it was evident that RagA/B in a GTP-bound state is responsible for determining mTORC1 activity instead of RagC/D. The studies also demonstrate that the GTP-bound state of RagA/B is regulated by amino acids (Figure 2). However, RagC/D (when it was co-expressed) also plays an important role for mTORC1 activation by enhancing the expression of the RagA/B mutant and by strengthening RagA/B through dimer formation. The maximal activity of mTORC1 resulted in the complete depletion of amino acids when GTP-bound RagA/B (dominant active) and GDP-bound RagC/D (dominant negative) were co-expressed. On the other side, the activity of mTORC1 is strongly suppressed even in amino acid-enriched conditions when co-expression of GDP bound RagA/B (dominant negative) and GTP-bound RagC/D (dominant active) takes place (Figure 2).

## 7. Regulation of Nutrient-Mediated Signalling at the Epigenetic Level

“Nutriepigenomics” involves the study of nutrients and their effects on human health via epigenetic modifications in gene expression without alteration in the DNA sequence. Although the DNA sequence is fairly permanent, epigenetic modifications are dynamic and heavily subjective to changes in external factors such as diet, lifestyle, and environment [118,119]. Chromatin remodelling, histone tail, and DNA modifications are examples of epigenetic modifications that have recently been extended to non-coding RNA and microRNA gene regulation [120]. A very well-known instance of nutriepigenomics is the progression of a honeybee into a queen or worker having identical genomes but with a difference in feeding, i.e., on royal jelly or a diet of pollen and nectar, respectively.

**Figure 3:**
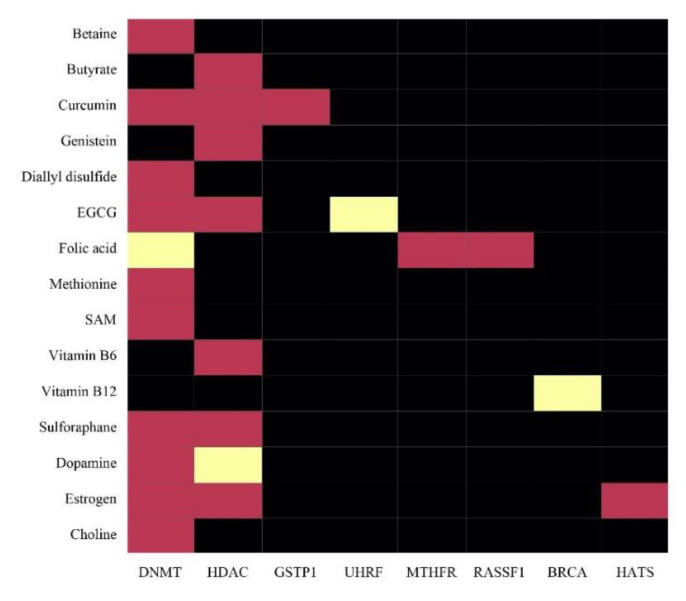
Heat map showing the effect of different nutrients on the expression of epigenetic enzymes. Red, yellow, and black colours indicate downregulation, upregulation, and no studies to date for genes expression, respectively. DNMT: DNA methyl transferase; HDAC: histone deacetylases; UHRF1: ubiquitin-protein transferase 1; MTHFR: methylenetetrahydrofolate reductase; RASSF1A: Ras association domain family protein1 isoform A; BRCA1: breast Cancer gene 1; HMTs: histone methyl transferases; HATs: histone acetyl transferases.

Nutrition influences epigenetic processes on multiple levels, i.e., DNA methylation, to regulate expression, and histone modifications alter localized DNA compaction. Early-life nutrition stimulates long-term modifications in DNA methylation, which further impacts an individual’s health and age-linked ailments throughout one’s life [119]. Nutrients either directly suppress the production of epigenetic enzymes, i.e., DNMT, HDAC, and HAT, or change the substrate availability essential for enzymatic reactions, thus impacting overall health and longevity [121] (Figure 3).

Nutrients work as methyl groups’ source or coenzymes for one-carbon metabolism regulating methyl group transfer. In DNA and histone methylation, B-vitamins, i.e., folate, vitamin B12, B6, and B2, work as coenzymes and methionine, choline, betaine, and serine as methyl group donors. Nutrients and bioactive food components immediately impact enzymes that catalyse DNA methylation and histone modifications. Diet has an influential role in the modification of the cellular milieu and thus causes alterations in epigenetic patterns [122].

The most-studied type of epigenetic modification is DNA methylation. DNA methylation, followed predominantly by dietary practices and intake of micronutrients, severely affects disease phenotypes. Nutrition influences patterns of DNA methylation in three ways: (i) substrates required for appropriate DNA methylation; (ii) cofactors that directly alter the enzymatic activity of DNA methyltransferases (DNMT); (iii) change in the activity of the enzymes mediating the one-carbon cycle. In the one-carbon metabolism pathway, methionine is converted into S-adenosylmethionine (SAM), with ATP as the substrate (Table 2).

Methylation of DNA by DNMTs involves covalent binding of SAM methyl groups to the cytosine bases (carbon-5 position), thus synthesizing 5-methylcytosine [131]. The precursors, such as methionine, folate, choline, betaine, and vitamins B2, B6, and B12, contribute to the methionine pathway at different sites, ultimately resulting in the net synthesis of SAM. Diets enriched with high-folate foods and fortified or supplemented with cofactors of one-carbon metabolism, such as folate and B12, directly play a crucial role in histone modifications [130].

Lower SAM synthesis and global DNA hypomethylation are the results of lesser availability of methyl donors and vice versa. Another crucial enzyme in one-carbon metabolism is methylenetetrahydrofolate reductase (MTHFR), which catalyses the biosynthesis of homocysteine to methionine and, therefore, results in the formation of 5- methyltetrahydrofolate. DNA re-methylation by dietary re-supplementation with folate in various food products has been successful globally. These strength-of-association outcomes are best seen in individuals with MTHFR mutations, depicting the crucial role of folate supplementation in the maintenance of methylation in persons suffering from MTHFR polymorphisms [126].

One of the most extensively researched micronutrients in various epidemiological DNA methylation studies is dietary folate, which acts as a single carbon donor, i.e., 5-methyl tetrahydrofolate (THF) reducing dihydrofolate (DHF) to THF. Accordingly, 5-methyl THF donates its methyl group to homocysteine, which forms methionine in the one-carbon metabolism cycle. Additionally, these transformations are facilitated by cofactor B-vitamins for enzymatic support—making easy the entry of dietary folate to replenish cellular SAM—in the one-carbon metabolism cycle. In the folate cycle, vitamin B6 acts as a coenzyme to serine hydroxymethyltransferase, a vital enzyme that converts THF to 5,10-methylene THF. Riboflavin acts as a precursor and cofactor for flavin adenine dinucleotides (FAD) and MTHFR, respectively. Vitamin B12 is a coenzyme of methionine synthase, catalysing the homocysteine reaction, which is the by-product of SAM synthesis to methionine (Table 2). As a result, the consumption of water-soluble B vitamins in the diet significantly impacts the efficiency of the one-carbon metabolism pathway [128]. Therefore, increased DNA methylation and folate restriction are related to folate intake [132].

Folate metabolism is correlated with phenotypic changes via DNA methylation because folate is a one-carbon source for synthesizing S-adenosylmethionine, required for DNA methylation [133]. The influential role in catalysing the methyl donor chemical reaction of epigenetically active constituents, i.e., folic acid and vitamin B12, affects overall DNA metabolism and DNA methylation maintenance patterns. Diets enriched with fruits and vegetables are enriched with natural antioxidants that protect against various diseases such as cancer. Methionine is an essential amino acid and acts as the precursor to SAM by its regular restoration from homocysteine in one-carbon metabolism. For the full activation of DNMT, SAM as a cofactor is required. A diet containing the methyl donors modulates the intracellular concentration of SAM, thus altering DNMT activity. The modulation of SAM pools indirectly regulates DNA methylation patterns; however, several compounds unswervingly affect DNMT expression or activity [134]. Myricetin decreases DNA methylation by inhibition of SssI DNMT. EGCG re-expresses several transcriptionally silenced genes via inhibiting DNMT1 activity, decreases growth, induces apoptosis in renal cell carcinoma by the re-expression of tissue factor pathway inhibitor-2 (TFPI-2), and decreases promoter hypermethylation. Further, dietary phenolic bioactives such as hesperetin, naringin, apigenin, and luteolin alter DNA methylation via indirect regulation of DNMT activity and SAM and SAH ratio alteration. Curcumin affects global hypomethylation in the MV4Nu–11 leukaemia cell line by suppressing DNMT [126].

## 8. Conclusions

Present lifestyles are enriched with the shift in the dietary system, eating habits, availability of diversified food, improved work standard, and increased economic status without compromising health. Luckily, the past few decades have been an eye-opener with the ability to study the human genome, which in turn has helped nutritional and medical researchers to address health-related challenges. Nutrigenomics-based studies have lifted the hopes of an unhealthy population, and it has become popular among a health-conscious population to use the information for maintaining current well-being and to work on future health plans via diet modification.

Nutrients directly regulate metabolic processes and impact the expression of hormones, receptors, and other proteins. An overall outlook on nutrient sensing mechanisms involves addressing potential nutrient sensing pathways and their cross-regulation. In the face of extensive research, our knowledge of the diverse mechanisms involved in nutrient sensing is far from complete. Future research is required to achieve a unified view of nutrient sensing mechanisms, which are essential for designing better supplements against various diseases.

“Nutritional epigenetics” offers promising insight into mechanisms explaining the modulation of dietary factors in several critical developmental steps that affect growth and health. Further research is needed to better understand the interactions among micronutrients, which are the main components of one-carbon metabolism, and the ones indirectly influencing the sustainability of the cycle’s effectiveness. In conclusion, sustained nutriepigenetic research will fortify our understanding of the major biological pathways linked with diet and health.

## Figures and Tables

**Table 1:** Genes altered by nutrients and related diseases.

Gene	Nutrient	Related Diseases	References
NAT2	High protein, Vitamin A, folic acid	Gastric cancer	[26]
GSTM1, ADH	Alcohol intake	Colorectal	[26]
Aflatoxins (polluted grains)	CYP2E1	Liver	[26]
CYP2E1	Nitrosamines (fried potatoes)	Nasopharyngeal, stomach	[26]
APOA2	Fat (high intake)	Obesity, dyslipidaemia	[27]
APOA5	Fat (high intake)	Obesity, dyslipidaemia	[27]
APOE	Vit. B9, choline (deficit)	Non-alcoholic fatty liver	[28,29]
ChREBP	Sugar (high intake)	Insulin resistance	[30]
CYP7A1	Protein (low intake)	Dyslipidaemia	[31]
DAT	Fat (high intake)	Obesity	[32]
FASN	Sugar and fat (high intake)	Non-alcoholic fatty liver, obesity	[33]
FOXA1	Vit. B9 and choline (deficit)	Non-alcoholic fatty liver	[28,29]
FOXA2	Vit. B9 and choline (deficit)	Non-alcoholic fatty liver	[28,29]
FTO	Protein (high intake)	Obesity	[34]
GATA4	Vit. A (deficit)	Cardiovascular diseases	[35]
HSD11B1	Calcium (deficit)	*Diabetes mellitus* (Type 2)	[36]
HSD11B2	Magnesium (deficit)	*Diabetes mellitus* (Type 2)	[37]
ICAM1	Selenium (deficit)	Cardiovascular diseases	[38]
Insulin signalling genes	Chromium (deficit)	*Diabetes mellitus* (Type 2)	[39]
LEP	Sugar and fat (high intake)	Obesity	[40]
MTHFR	Vit. B9 (low intake)	Cardiovascular diseases, cancer	[41,42]

NAT2: N-acetyl transferase 1,2; GSTM1: glutathione-S-transferase μ−1; ADH: antidiuretic hormone; CYP2E1: cytochrome P450 2E1; APOA2: apolipoprotein A-II; APOA5: apolipoprotein 5; APOE: apolipoprotein E; ChREBP: carbohydrate response element-binding protein; CYP7A1: cholesterol 7α-hydroxylase; DAT: dopamine transporter; FASN: fatty acid synthase; FOXA1: Forkhead box protein A1; FOXA2: Forkhead box protein A2; FTO: fat mass and obesity-associated protein; GATA4: GATA binding protein 4; HSD11B1: 11β-Hydroxysteroid dehydrogenase type 1; HSD11B2: 11β-Hydroxysteroid dehydrogenase type 2; ICAM1: intercellular adhesion molecule 1; LEP: lysosomal enzyme-rich preparations; MTHFR: methylene-tetrahydro-folate reductase.

**Table 2:** Dietary components contribute to providing protection against cancers and their mechanism of action.

Nutrient	Food Origin	Epigenetic Role	References
Betaine	Wheat, spinach, sugar beets	Break down the toxic by-products of SAM synthesis	[123]
Butyrate	An intestinal compound	Increased histone acetylation turning on “protective” genes	[124]
Choline	Egg yolks, cooked beef, chicken	Methyl donor to SAM	[125]
Curcumin	*Curcuma longa*	Regulation of DNMT and SAM synthesis	[122]
Diallyl sulphide	Garlic	Increased histone acetylation turning on anticancer genes	[118]
EGCG	Green tea polyphenol	DNMT1 inhibition	[126]
Genistein	Soybean	SAM synthesis, increased methylation	[126]
Folic Acid	Leafy vegetables, sunflower seeds, baker’s yeast	Methionine synthesis	[127]
Methionine	Sesame seeds, Brazil nuts, peppers, spinach	SAM synthesis	[128]
SAM-e (SAM)	Popular dietary supplement pill	Enzymes transfer methyl groups from SAM to the DNA	[120]
Vitamin B6	Meats, whole grain products, vegetables	Methionine synthesis	[128]
Vitamin B12	Meat, liver, shellfish, milk	Methionine synthesis	[128]
Sulforaphane	Broccoli	Increased histone acetylation turning on anticancer genes	[129]
Dopamine	Amino acid tyrosine	Role in reward and movement regulation	[130]
Oestrogen	Dairy, nuts and seeds, legumes	Epigenetic transcription factor JAK2	[130]
